# Improving beef quality through rumen microbial modulation by varying alfalfa hay proportions in the diet

**DOI:** 10.3389/fmicb.2026.1961267

**Published:** 2026-09-29

**Authors:** Boshuai Liu, Yujie Zheng, Zimin Gao, Defeng Li, Xiaoyan Zhu, Hao Sun, Zhichang Wang, Yalei Cui, Yinghua Shi

**Affiliations:** 1 Department of Animal Nutrition and Feed Science, College of Animal Science and Technology, Henan Agricultural University, Zhengzhou, Henan, China; 2 Henan Key Laboratory of Innovation and Utilization of Grassland Resources, Zhengzhou, Henan, China; 3 Henan Herbage Engineering Technology Research Center, Zhengzhou, Henan, China

**Keywords:** alfalfa hay, beef cattle, growth performance, meat quality, microbiota, rumen

## Abstract

**Introduction:**

This study evaluated the effects of different dietary proportions of alfalfa hay on growth performance, carcass characteristics, meat quality, rumen fermentation, and rumen microbiota in beef cattle.

**Methods:**

Sixty Simmental crossbred cattle were randomly assigned to diets containing 11.57% (low alfalfa hay group, LAG), 20.83% (medium alfalfa hay group, MAG), or 30.09% (high alfalfa hay group, HAG) alfalfa hay, with four replicates per treatment and five cattle per replicate.

**Results:**

The LAG diet significantly increased final body weight and reduced beef shear force. The LAG diet also resulted in the highest saturated fatty acid content in the longissimus thoracis muscle. Based on 16S rRNA analysis, the LAG group had significantly higher relative abundances of Eubacterium_brachy_group and norank_f_Eubacterium_coprostanoligenes_group. Muscle n-6 polyunsaturated fatty acid content was positively correlated with the relative abundance of Christensenellaceae_R-7_group, whereas Succiniclasticum abundance was negatively correlated with the PICRUSt2-predicted acarbose and validamycin biosynthesis pathway.

**Discussion:**

A dietary alfalfa hay proportion of 11.57% improved final body weight and beef tenderness and was associated with distinct changes in rumen microbial composition and predicted microbial functions. These findings provide a scientific basis for using alfalfa hay to produce high-quality beef.

## 1 Introduction

Over the past 40 years, the Chinese population's increasingly complex dietary habits, coupled with enhanced living standards have brought about an amazing rise in beef consumption, market research has indicated that consumers are willing to pay a premium for high-quality beef options (Li et al., 2018). Paradoxically, the Chinese beef industry's reliance on inexpensively sourced roughage such as wheat and corn straw has emerged as a formidable challenge. The beef produced from these substandard roughages has compelled consumers to exhibit an increasing interest in imported beef due to its higher quality. Addressing this increasingly perplexing challenge by adopting the use of high-quality forage has become an urgent and complex issue that requires immediate attention.

Diet is a key factor affecting animal products, nutrients in diets directly affect beef quality. Alfalfa is a highly regarded feed option that is extensively utilized in ruminant nutrition, specifically for feeding dairy cattle, alfalfa is a good source of protein, fiber, and various other nutrients, feeding alfalfa hay can improved feed efficiency, increased milk yield. In the period before weaning, supplementation with alfalfa hay has been shown to enhance the development of the rumen's epithelium and muscles in dairy calves (Norouzian et al., 2011). Moreover, including alfalfa hay in the diet has been found to elevate the number of cellulose-degrading bacteria in the rumen. and thus improve both rumen health and development of the rumen epithelium. Zhang et al. (2022a) indicated that beef finished with alfalfa had better physical and organoleptic quality than beef finished with millet or fescue, finishing on alfalfa hay increased the concentration of linolenic acid. Pordomingo et al. (2021) found that the sensory panel appreciates finished beef on alfalfa pasture over winter annual grasses, and alfalfa finished beef has greater n-3 PUFA than beef finished on winter annual grasses.

The rumen is an intricate and constantly changing environment, housing anaerobic microorganisms such as bacteria, protozoans, fungi, archaea, and viruses that interact with each other to obtain energy by breaking down the cellulose in plant cell walls. The composition of this microbial community is primarily influenced by dietary factors. typically, feedlot operations employ diets high in grain content to maximize animal growth rates and minimize the negative effects of low dietary protein levels (Zhang N. et al., 2022), this type of diet often results in reduced bacterial diversity and lower concentrations of microbes that are capable of breaking down fiber. Moreover, high grain diet causes acidosis due to a rapid increase in VFAs concentration and a sudden decrease in rumen pH. Compare with high grain diets, high grass diets provide an optimum dietary content of fiber to ensure rumen health and high efficiency, and several studies have found that high grass diets markedly increased the omega-3 fatty acid concentration of beef relative to beef conventionally finished on high grain diet (Zhang et al., 2022b).

Therefore, the aim of this study is to delve into the influence of different proportions of alfalfa hay in the daily feed on the rumen microbial community and to ascertain the most favorable ratio of alfalfa hay in the diet. The outcomes of this study will provide insights into the practical application of alfalfa hay for the production of superior quality beef.

## 2 Materials and methods

### 2.1 Animals diet and experimental design

Sixty Simmental hybrid cattle (415 kg, 18 months old) were divided into three dietary groups, each with four replicates (five cattle per replicate). A 7-day adaptation period preceded the experiment. Cattle were fed total mixed ration (TMR) twice daily. Standard feeding and immunization protocols were followed. All animals were managed identically. Feed ingredients and detailed nutritional composition, including calculated net energy for maintenance (NEmf), crude protein (CP), neutral detergent fiber (NDF), and acid detergent fiber (ADF) contents, are fully provided in Supplementary Table S1. This ensures that reviewers and readers can confirm that physiological responses are attributable specifically to varying alfalfa inclusion proportions rather than confounding energy or protein imbalances. Initial and final body weights were recorded before morning feeding.

### 2.2 Sample collection

Forty-eight hours before slaughter, 50 ml rumen fluid samples were collected from one animal per replicate. The fluid was frozen in liquid nitrogen and stored at −80 °C for bacterial diversity analysis. Animals were transported to Hengdu Commercial Abattoir in Henan, China, 1 day before slaughter. All animals were stunned and exsanguinated. Body weight and pH at 45 min post-slaughter were recorded using a portable pH meter. Carcasses were aged at 4 °C for 48 h. A 2.5 cm steak was collected from the left posterior longissimus thoracis (LT) before aging, and pH at 48 h was recorded.

### 2.3 Meat quality measurements

Aged muscle samples were analyzed for ash, crude protein, moisture, and fat content. Drip loss was measured after 24 h at 4 °C. Shear force (Warner-Bratzler shear apparatus) and cooking loss were determined following standard procedures.

### 2.4 Fatty acid analysis

For fatty acid analysis, 1 g of muscle tissue was methylated. Methyl esters were quantified by gas chromatography (GC/MSD; Agilent) with a flame-ionization detector and a fused silica capillary column. The GC program ramped from 80 to 230 °C.

### 2.5 Rumen fermentation parameters

Rumen fluid pH was measured with a Sartorius PB-10 pH meter. Short-chain fatty acids were quantified by gas chromatography (GC) using a Trace 1310 GC with a flame ionization detector and HP-88 column. The temperature program started at 70 °C and increased to 240 °C.

### 2.6 Rumen bacterial diversity analysis

DNA was extracted using the E.Z.N.A. Soil DNA Kit. DNA quality was assessed by spectrophotometry and electrophoresis. PCR amplification of the 16S rRNA gene was performed with primers 338F and 806R. Products were purified, quantified, and sequenced on an Illumina MiSeq platform.

### 2.7 Bioinformatics analysis

Raw fasta files were filtered using Trimmomatic and merged with FLASH. Sequences were clustered at a 97% similarity cutoff using UPARSE. Biodiversity was assessed using ACE, Chao1, and Shannon indices. Data were analyzed with R software. Principal coordinate analysis (PCoA) and redundancy analysis (RDA) were used to evaluate microbiota interactions. PICRUSt2 was used to predict metabolic pathways. PICRUSt2 was used to computationally infer the potential functional profiles of the rumen bacterial communities from 16S rRNA gene sequencing data. Because PICRUSt2 provides predicted rather than directly measured functional information, the resulting pathway profiles were interpreted as computational inferences and not as direct measurements of microbial gene content or metabolic activity.

### 2.8 Statistics and analysis

Statistical analyses were performed using a linear mixed-effects model. Dietary treatment was included as a fixed effect. For growth performance, carcass characteristics, and meat-quality traits measured at the individual-animal level, pen was included as a random effect to account for the non-independence of animals housed within the same replicate pen. For rumen fermentation and microbial measurements, one animal was sampled from each replicate pen; therefore, the replicate pen/animal was considered the experimental unit, resulting in four independent observations per treatment. When the overall treatment effect was significant, pairwise comparisons among treatments were performed using an appropriate multiple-comparison procedure. Data are presented as means with the corresponding standard error of the mean (SEM). Statistical significance was declared at *P* < 0.05, whereas 0.05 ≤ *P* < 0.10 was interpreted only as a statistical tendency. Different superscript letters within a row indicate significant differences among treatment means at *P* < 0.05.

## 3 Results

### 3.1 Growth performance and carcass characteristics

The final weight was different between LAG, MAG and HAG (Table 1), LAG diet significantly increased the final weight and ADFI of Simmental crossbred cattle (*p* < 0.05), ADG and F/G were not significantly affected by increasing the proportion of alfalfa hay in the diet (*p* > 0.05). There were no significant differences (*p* > 0.05) in cold carcass weight, dressing percentage, net meat mass, net meat percentage, meat mass/bone mass, and loin-eye area among the different dietary alfalfa proportions.

**Table 1:** Performance, carcass characteristics, chemical composition, and meat quality of the longissimus thoracis muscle in Simmental crossbred cattle.

Items	LAG	MAG	HAG	SEM	*P*-value
Growth performance					
Initial weight/kg	423.28	403.51	414.73	13.78	0.224
Final weight/kg	621.53^a^	585.91^b^	564.16^b^	20.87	0.041
ADFI/kg/d	10.78^a^	10.99^a^	9.28^b^	0.83	0.001
ADG/kg/d	1.98	1.90	1.75	0.22	0.443
F/G	5.43	5.77	5.39	0.75	0.217
Carcass characteristics					
Cold carcass weight, kg	344.25	320.84	319.62	13.06	0.507
Dressing percentage, %	54.39	55.54	55.73	0.52	0.645
Net meat mass, kg	310.57	287.95	286.63	14.88	0.486
Net meat percentage, %	49.64	49.98	49.96	0.12	0.734
Meat mass/Bone mass	9.16	9.00	8.67	0.15	0.259
Loin-eye area, cm	130.55	138.25	142.42	4.77	0.249
Chemical compositions					
Moisture %	73.93	73.84	73.08	0.11	0.119
Crude protein %	22.45	21.79	22.20	3.77	0.080
Fat %	4.49	4.98	4.97	0.40	0.484
Ash %	1.12	1.02	1.19	3.27	0.142
Meat quality					
Cooking yield, %	58.33	60.29	59.21	3.91	0.387
Shear force (*N*)	81.27^b^	104.01^ab^	114.22^a^	7.75	0.043
Drip loss at 24 h, %	3.53	3.26	3.94	0.008	0.253
pH45min	5.58	5.67	5.59	0.17	0.225
pH48	6.29	6.06	6.78	0.62	0.186

Different superscript letters indicate significant differences at *P* < 0.05.

### 3.2 Meat quality

As shown in Table 1, The LAG diet significantly reduced shear force in beef (*p* < 0.05), moisture, crude protein, ash, fat contents of meat, cooking yield, drip loss at 24 h, pH45 min, and pH48 were not significantly influenced by three different dietary alfalfa proportion diets (*p* > 0.05).

### 3.3 Fatty acids composition

The fatty acid composition of the LT muscle is presented in Table 2. The C16:1 content showed a numerical tendency to differ among dietary treatments (*p* = 0.059), but the difference did not reach the pre-defined threshold for statistical significance. The proportions of C17:0 and SFA decreased significantly with increasing dietary alfalfa hay inclusion (*p* < 0.05). In contrast, C20:1 increased significantly with increasing dietary alfalfa hay inclusion (*p* < 0.05).

**Table 2:** The effects of different dietary forage combination on fatty acids in simmental crossbred cattle muscle.

Items (% of total fatty acid)	LAG	MAG	HAG	SEM	*P*-Value
C14:0	2.05	2.63	2.09	0.04	0.119
C14:1	0.29	0.64	0.42	0.03	0.167
C15:0	0.34	0.35	0.26	0.01	0.118
C16:0	28.37	28.27	27.63	0.10	0.608
C16:1	2.41	3.74	2.97	0.08	0.059
C17:0	0.90^a^	0.75^b^	0.73^b^	0.01	0.023
C17:1	0.54	0.55	0.53	0.01	0.706
C18:0	20.33	16.99	17.84	0.22	0.085
C18:1	40.52	41.84	42.92	0.19	0.229
C18:2n6	2.83	2.86	3.00	0.04	0.873
C18:3n6	0.10	0.09	0.08	0.01	0.249
C18:3n3	0.19	0.19	0.22	0.01	0.564
C20:0	0.13	0.11	0.11	0.01	0.233
C20:1	0.17^b^	0.18^b^	0.22^a^	0.01	0.040
C20:3n3	0.47	0.48	0.63	0.01	0.244
SFA	52.14^a^	49.09^ab^	48.65^b^	0.22	0.042
MUFA	43.93	46.96	47.06	0.22	0.078
PUFA	3.60	3.62	3.93	0.05	0.683
n−6 PUFA	2.85	2.86	2.86	0.30	0.305
n−3 PUFA	0.58	0.59	0.59	0.07	0.070
n−6 PUFA/n−3 PUFA (%)	5.27	4.91	4.91	0.62	0.616

SFA, saturated fatty acids (14:0+ 15:0 + 16:0 + 17:0 +18:0 + 20:0);

MUFA, monounsaturated fatty acids (14:1 +16:1 + 17:1 + 18:1 + 20:1);

PUFA, polyunsaturated fatty acids (18:2n−6 + 18:3n−3 + 18:3n−6 + 20:3n−3);

n−6 PUFA (C18:2n6 + 18:3n−6);

n−3 PUFA (18:3n−3 + 20:3n−3);

^a, b^ means within rows with different superscript letters differ (*P* < 0.05).

### 3.4 Rumen fermentation

The results of rumen fermentation are listed in Table 3. In general, compared with the MAG group, the HAG diet significantly reduced the acetic acid concentration in the rumen (*p* < 0.05), and there were no significant differences observed in the content of NH3-N, propionate, butyrate, isobutyric acid, valeric acid, and isovaleric acid among the three groups (*p* > 0.05). Additionally, the increasing dietary alfalfa hay proportion had no effect on pH and A/P-value (*p* > 0.05).

**Table 3:** Rumen fermentation parameters in Simmental crossbred cattle.

Items	LAG	MAG	HAG	SEM	*P*-value
pH	6.51	6.43	6.41	0.69	0.382
NH_3_-N/mg/dl	38.81	36.20	49.35	7.87	0.334
Acetate/mg/ml	35.67^ab^	37.97^a^	29.01^b^	5.17	0.050
Propionate/mg/ml	12.66	14.35	10.99	3.13	0.425
Butyrate/mg/ml	9.07	12.02	7.87	2.74	0.150
Isobutyric/mg/ml	0.29	0.41	0.24	0.11	0.105
Valeric/mg/ml	0.87	1.11	0.80	0.04	0.667
Isovaleric/mg/ml	0.44	0.62	0.46	0.12	0.245
A/P	2.75	2.69	2.69	0.18	0.317

Different superscript letters indicate significant differences at *P* < 0.05.

### 3.5 Rumen microbes

After amplification by the Illumina HiSeq sequencing system, a total of 1,165,785 16S rRNA gene sequences of high quality were obtained. However, more than 5.5% (58,893 sequences) were identified as chimeric and were excluded from further analysis, the mean sequence read length was 414 bp. Finally, 576,848 qualified sequences and 2,631 clean OTUs were subjected to further analyses. There were no significant changes observed in Chao, Sobs, and Shannon index at the OTU level in the alpha diversity analysis (Figures 1A–C, *p* > 0.05). Further beta diversity analysis revealed the microbial diversity of LAG, MAG and HAG were similar (Figure 1D).

**Figure 1:**
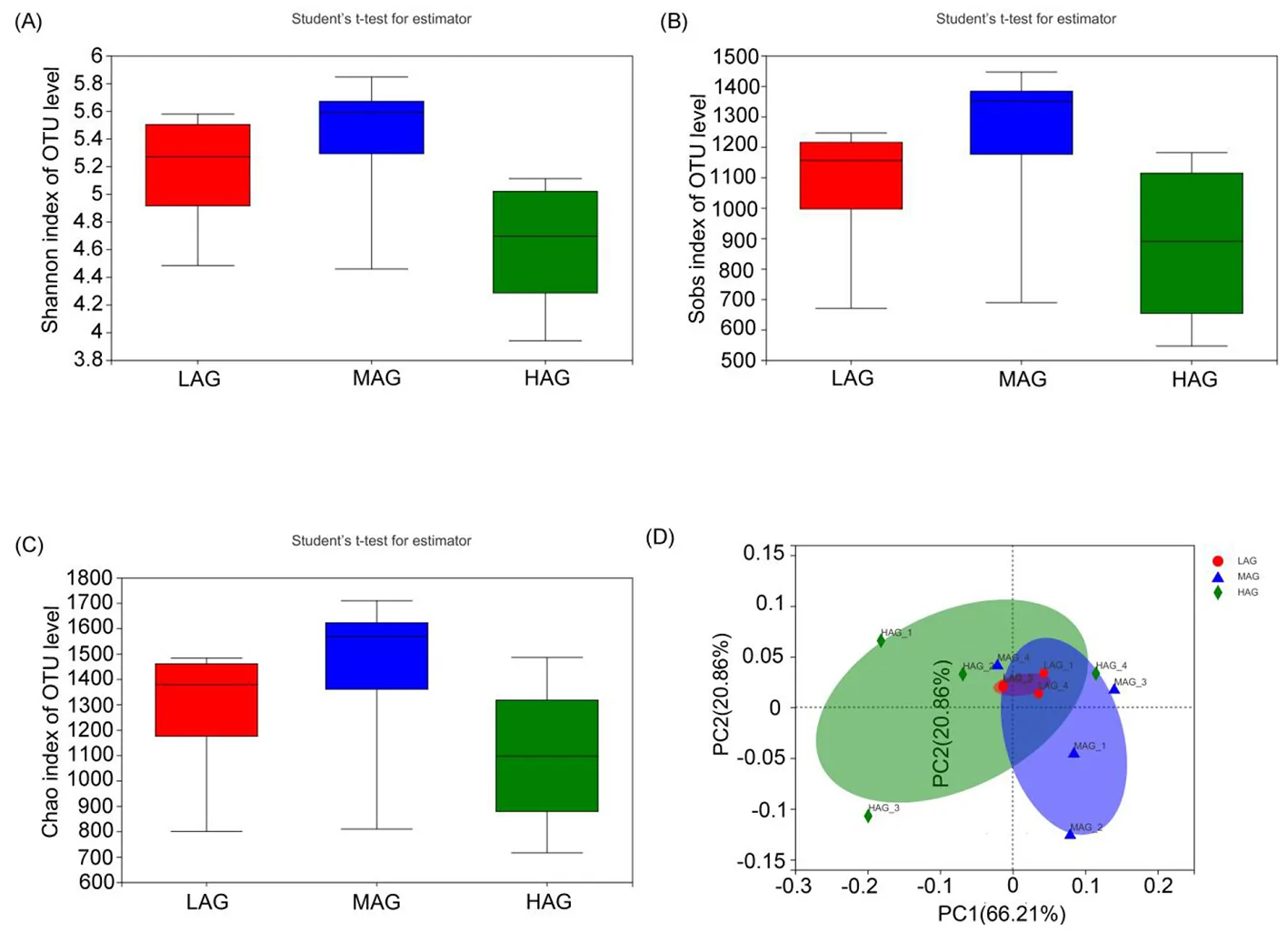
Alpha diversity statistics comparison. **(A)** Chao index at the OTU level. **(B)** Sobs index at the OTU level. **(C)** Shannon index at the OTU level. **(D)** Principal coordinate analysis (PCoA) of rumen bacterial community structures in the three treatment groups based on weighted UniFrac distances. Cattle were fed a low alfalfa hay diet (LAG), *n* = 4; a medium alfalfa hay diet (MAG), *n* = 4; or a high alfalfa hay diet (HAG), *n* = 4.

Figure 2 displays the community composition at both the phylum and genus levels. In three groups, the dominant microbiota at the phylum level were *Firmicutes* (LAG66.42%, MAG58.7%, HAG73.83%, Figure 2A), *Bacteroidetes* (LAG28.23%, MAG30.91%, HAG19.56%, Figure 2A). At the genus level, the microbiota was affiliated to 36 genera, dominant genera were *Prevotella* (LAG4.41%, MAG8.67%, HAG6.46%, Figure 2B), *Ruminococcus* (LAG7.89%, MAG4.84%, HAG9.62%, Figure 2B), *Rikenellaceae_RC9_gut_group* (LAG8.22%, MAG7.65%, HAG3.56%, Figure 2B), *NK4A214_group* (LAG6.75%, MAG6.23%, HAG5.63%, Figure 2B). Interestingly, increasing the dietary alfalfa hay proportion decreased the relative abundance of *Actinobacteriota* and increased the relative abundance of *Proteobacteria* (Figures 3A,B). Additionally, the relative abundance of *Eubacterium_brachy_group* was significantly higher in LAG than in HAG (*p* < 0.05) and MAG (*p* < 0.01), as shown in Figure 3C. Furthermore, the LAG group had a significantly higher relative abundance of *norank_f_Eubacterium_coprostanoligenes_group* than the HAG group (Figure 3D, *p* < 0.01). The changes in dietary alfalfa content also affected the relative abundance of various genera.

**Figure 2:**
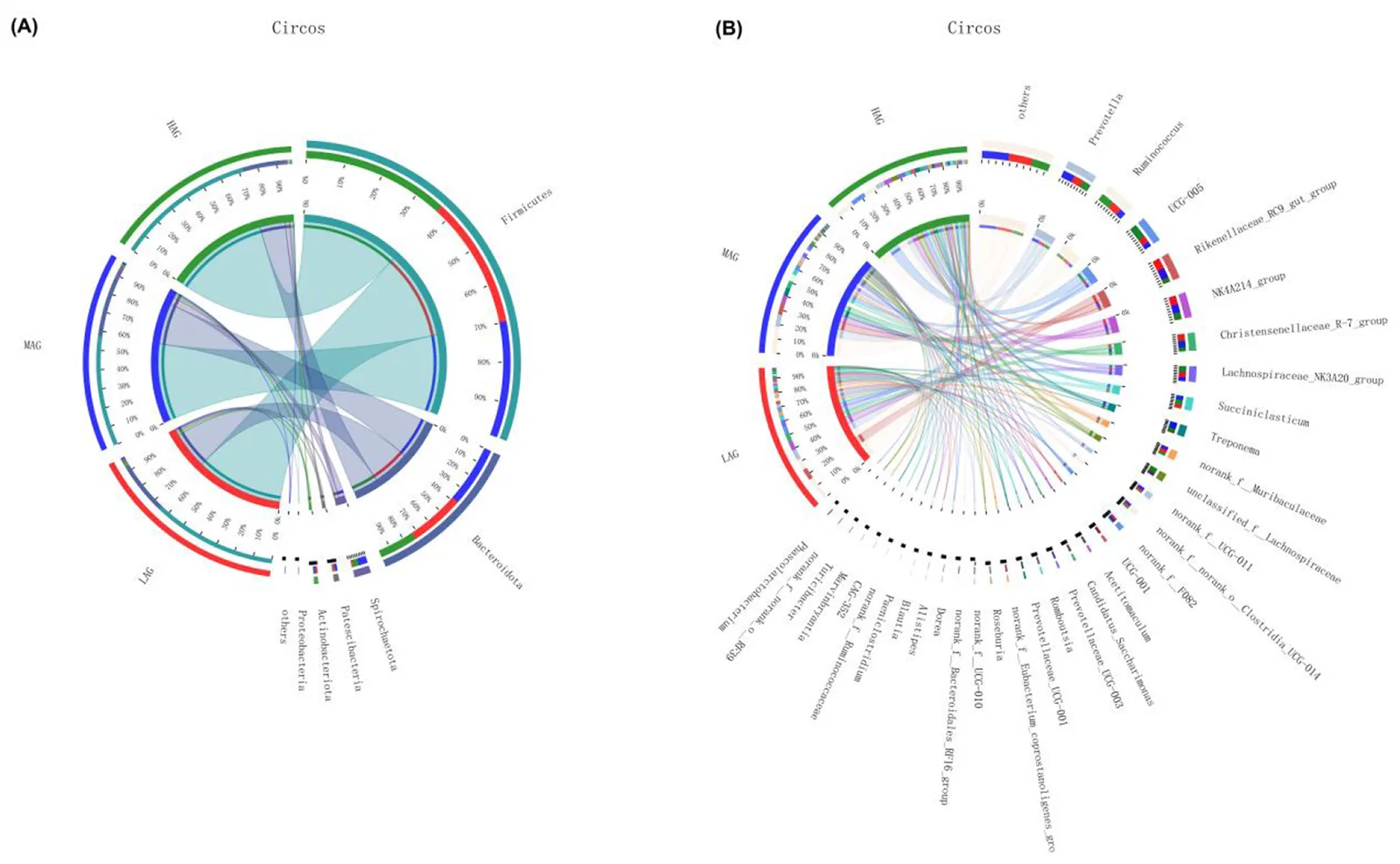
The relative abundance of phyla and genera in rumen bacteria community; for enhanced readability, the text size within the high-resolution figure panels has been enlarged to clearly differentiate the microbial groups. **(A)** Community abundance on phylum level in three treatment groups. **(B)** Community abundance on genus level in three treatment groups. Cattle were fed a low alfalfa hay diet (LAG), *n* = 4; Cattle were fed a medium alfalfa hay diet (MAG), *n* = 4; Cattle were fed a high alfalfa hay diet (HAG), *n* = 4.

**Figure 3:**
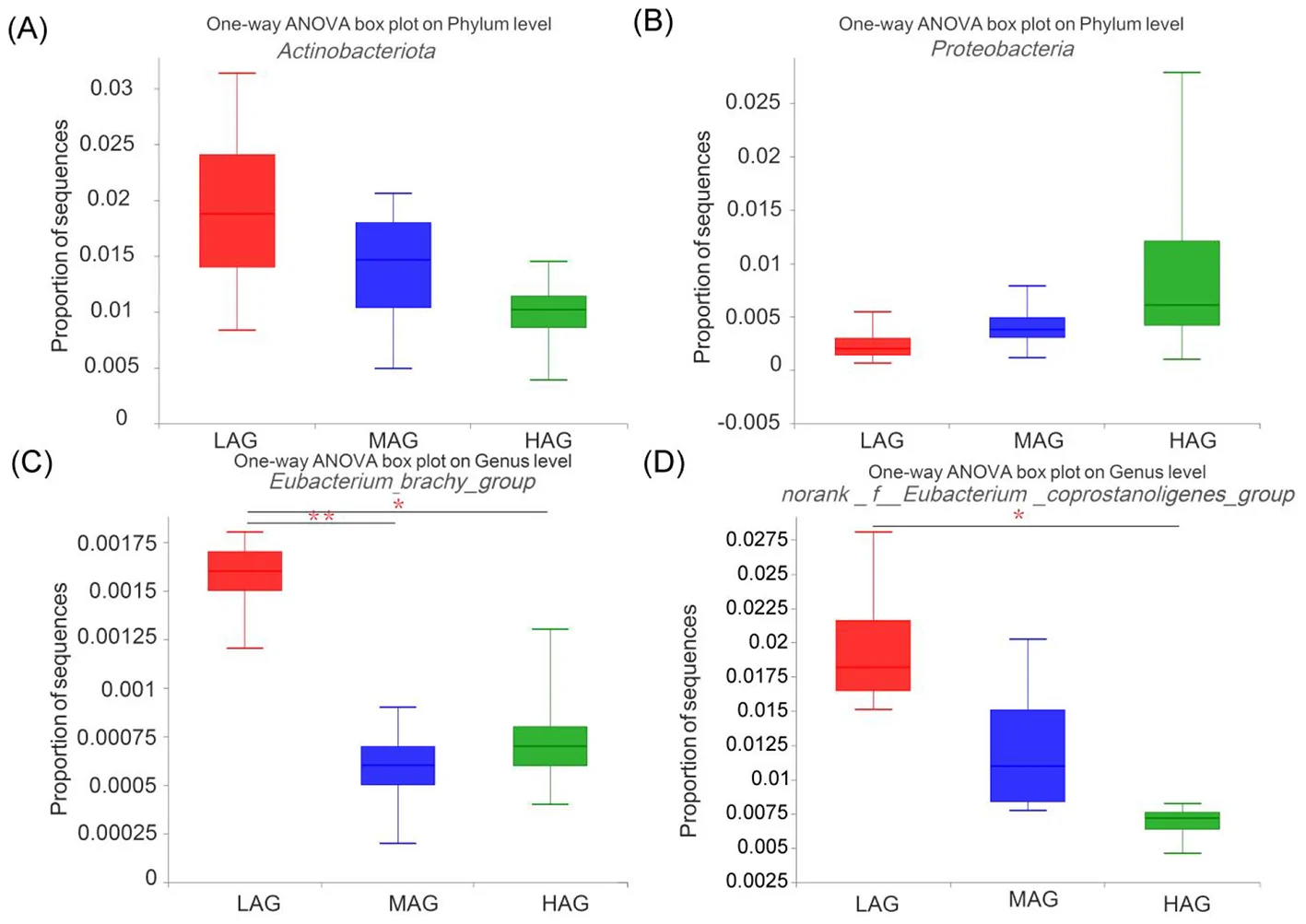
Difference of rumen bacteria at the phylum level and genus level. **(A)** Analysis on the difference of *Actinobacteriota* abundance at phylum level. **(B)** Analysis on the difference of *Proteobacteria* abundance at phylum level. **(C)** Analysis on the difference of *Eubacterium_brachy_group* abundance at genus level. **(D)** Analysis on the difference of *norank_f__Eubacterium_coprostanoligenes_group* abundance at genus level. LAG, low alfalfa hay group, *n* = 4; MAG, medium alfalfa hay group, *n* = 4; HAG, high alfalfa hay group, *n* = 4. *P < 0.05, **P < 0.01.

### 3.6 The correlation of bacterial genera between meat quality and VFA concentrations

To correlate the bacterial genera to muscle fatty acid deposition and rumen fermentation parameters, Spearman correlations were obtained independently for each breed. Only genera with a relative abundance of the top 15 were considered and only display significant correlations. According to Figure 4, *Prevotella* was positively correlated with the concentration of isovaleric acid. The concentration of acetic acid was positively correlated with *Rikenellaceae_RC9_gut_group*.

**Figure 4:**
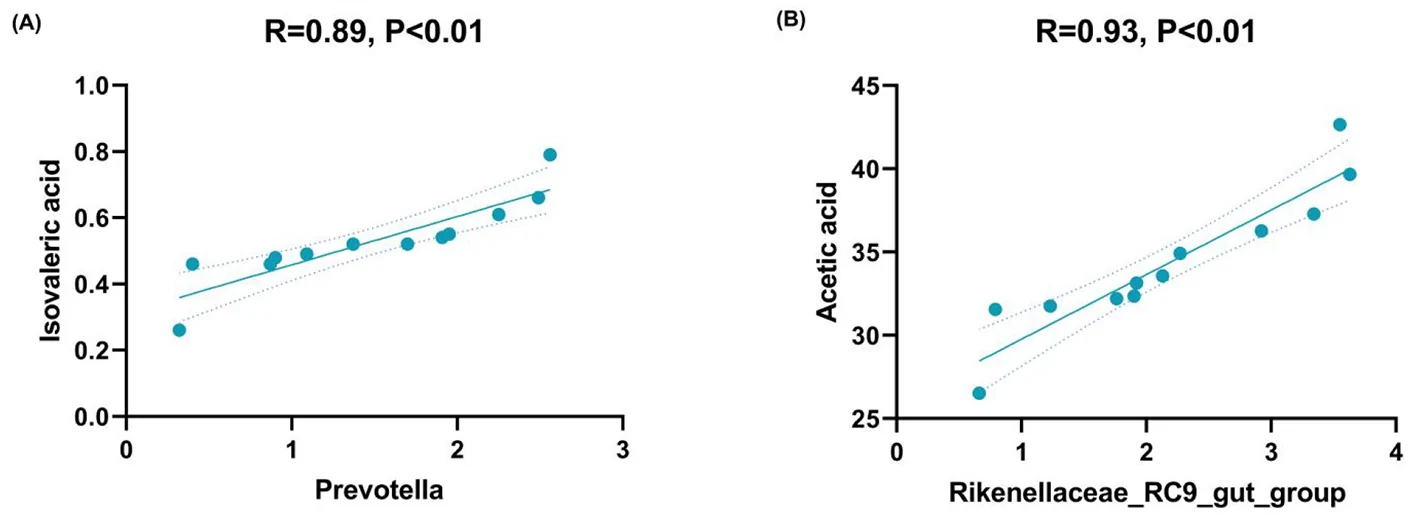
Correlation between rumen microorganism and environmental factors. Only display significant correlations. **(A)** The correlation between the relative abundance of *Prevotella* and the concentration of isovaleric acid in the rumen. **(B)** The correlation between the relative abundance of *Rikenellaceae_RC9_gut_group* and the concentration of acetic acid in the rumen.

### 3.7 The effects on the metabolic pathway

A total of 353 metabolic pathways were predicted. After filtering, 205 pathways were retained, of which the top 50 are shown in Figure 5A. Figure 5B shows the differential pathways, compared with MAG, the diet of the HAG group significantly increased the relative abundance of Biosynthesis of amino acids, Glycolysis/Gluconeogenesis, Glycine, serine and threonine metabolism, 2-Oxocarboxylic acid metabolism. These computational predictions indicate potential microbial shifts, but definitive causal relationships cannot be established unless backed by future metabolomic or transcriptomic data.

**Figure 5:**
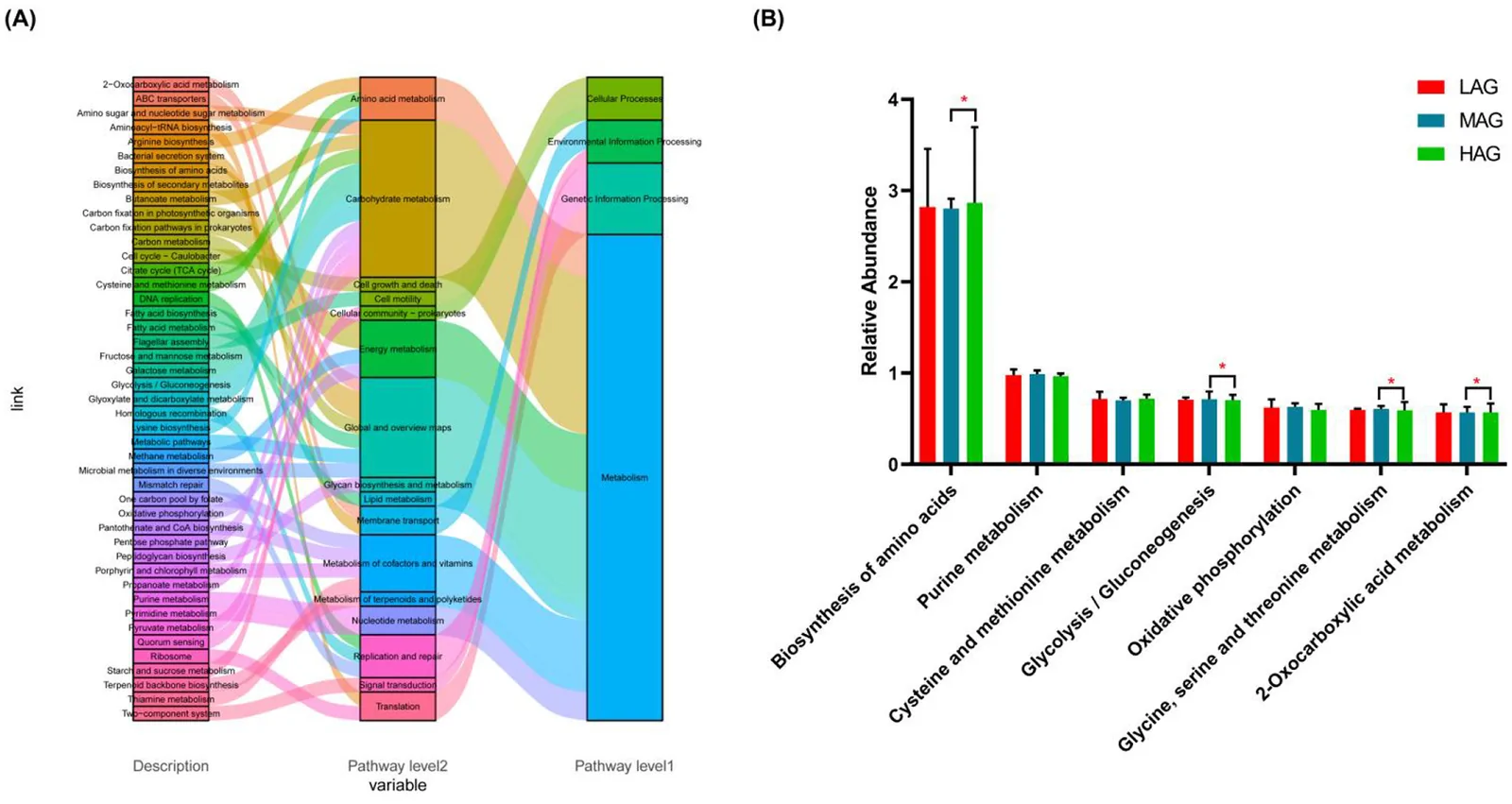
Prediction of KEGG pathways by PICRUSt2. **(A)** Top 50 metabolic pathways among the 205 pathways retained after filtering. **(B)** Analysis of differences in rumen microbial functions. *P < 0.05.

The correlation between rumen microorganisms and metabolic pathways showed in Figure 6, the abundance of *Treponema* was significantly positively correlated with Alanine, aspartate and glutamate metabolism, Pentose and glucuronate interconversions, Fructose and mannose metabolism, Nitrogen metabolism, Galactose metabolism, Streptomycin biosynthesis, Polyketide sugar unit biosynthesis. The abundances of *Ruminococcus* were significantly negatively correlated with Polyketide sugar unit biosynthesis and Acarbose and validamycin biosynthesis, the abundances of *NK4A214_group* were significantly negatively correlated with Acarbose and validamycin biosynthesis, Streptomycin biosynthesis, Galactose metabolism. The abundances of *NK4A214_group* also significantly positively correlated with D-Arginine and D-ornithine metabolism. The abundances of *Succiniclasticum* were significantly negatively correlated with Acarbose and validamycin biosynthesis. The abundance of *Succiniclasticum* also significantly positively correlated with D-Arginine and D-ornithine metabolism. Meanwhile, *norank_f__Muribaculaceae* were dramatically negatively correlated with the Biosynthesis of various secondary metabolites-part 1, Histidine metabolism, Biosynthesis of 12-, 14- and 16-membered macrolides, Taurine and hypotaurine metabolism, Biotin metabolism, Biosynthesis of type II polyketide backbone. The abundance of *norank_f__UCG-001* was positively correlated with Taurine and hypotaurine metabolism, Biotin metabolism. Conversely, the abundance of *UCG-005* was negatively correlated with Taurine and hypotaurine metabolism, Biotin metabolism. *Prevotella* was negatively correlated with Ether lipid metabolism. *Unclassified_f__Lachnospiraceae* had a negative correlation with Indole alkaloid biosynthesis, whereas *norank_f__ norank_o__Clostridia_UCG-014* had a positive correlation with Stilbenoid, diarylheptanoid, and gingerol biosynthesis.

**Figure 6:**
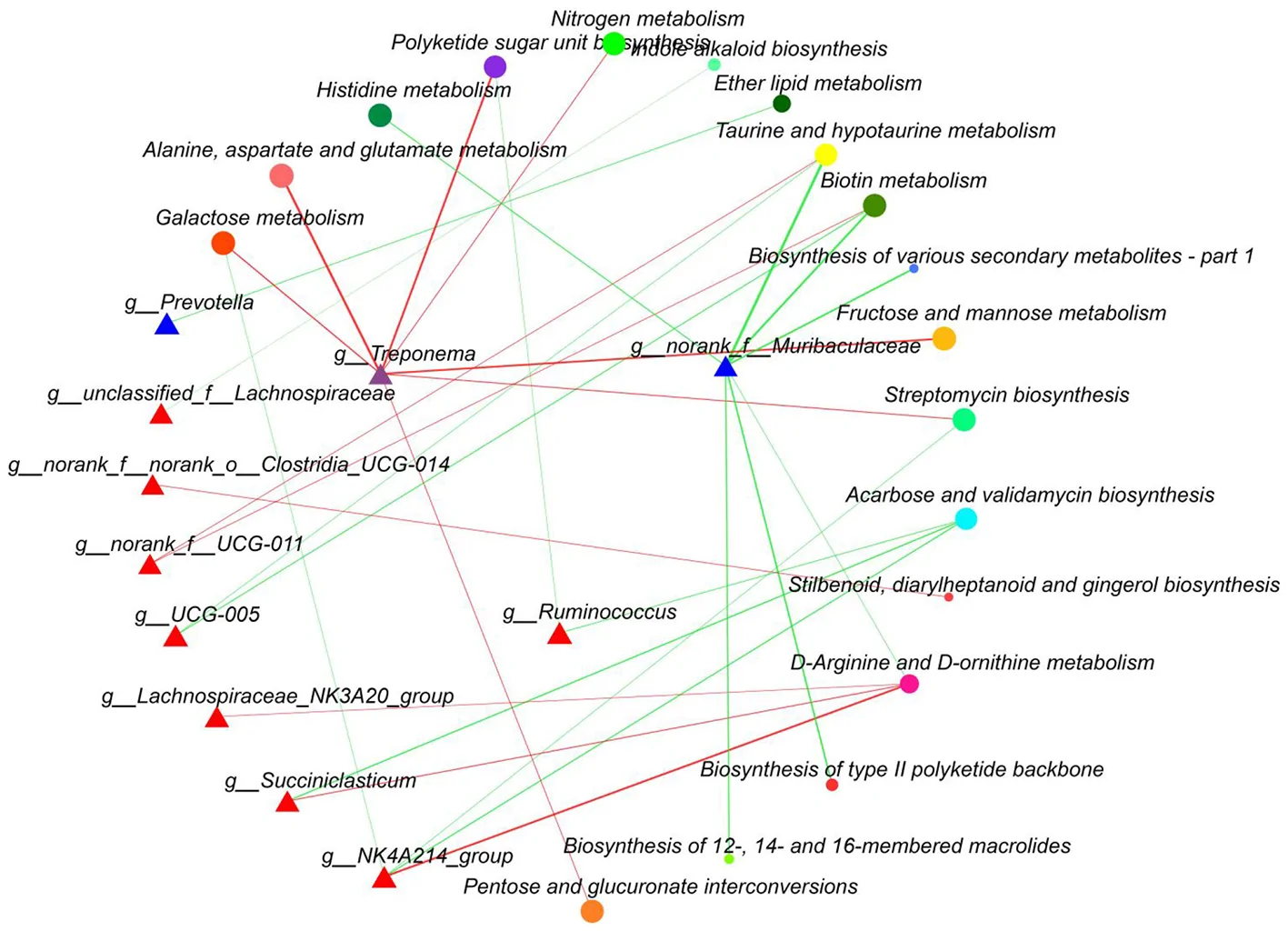
Correlations between rumen microorganisms and PICRUSt2-predicted KEGG pathways. Only significant correlations are displayed. Red lines indicate positive correlations, whereas green lines indicate negative correlations. Line thickness represents the magnitude of the correlation coefficient, with thicker lines indicating stronger correlations.

## 4 Discussion

Animal production performance evaluation is crucial, with average daily gain (ADG) being a key metric that reflects feed conversion, Simmental hybrid cattle mainly consume high-quality roughage as their main source of fiber and other essential nutrients. According to Madruga et al. (2018) exceeding 19% dietary content of alfalfa hay led to a significant reduction in daily gain in cattle, while Swanson et al. (2017) recommended not exceeding 10%. In this study, the HAG diet was found to decrease ADG, as well as final weight when compared to LAG and MAG diets. Carcass yield is an essential parameter for beef production, although increasing the proportion of dietary alfalfa hay did not affect carcass characteristics, cold carcass weight tended to decrease, possibly because the high content of neutral detergent fiber (NDF) and acid detergent fiber (ADF) is responsible for this effect. These components significantly affect nutrient digestibility. Deng et al. (2023) reported, high NDF and ADF intake resulted in decreased final body weight. The study suggests that optimal levels of alfalfa should not be exceeded in the diets of beef cattle, as high alfalfa content reduces production performance in beef cattle.

The content of fatty acids in beef is an important factor affecting the quality of beef. On the one hand, the fatty acid content in beef has a direct impact on its flavor, thermal oxidation of lipids during cooking was more readily in unsaturated fatty acids, the decomposition of fatty acid yield ketones, carboxylic acids, alcohols, and alkanes was the main source of flavor. Fatty acid content in beef reflects intensity of aroma after cooking. Zhang et al. (2023) reported that hexanal, derived from oleic acid, provided a greenish smell in cooked beef, while higher levels of linoleic acid produced octanal, giving off a soapy aroma after cooking. The healthier fatty acid content in beef can reduce the incidence of cardiovascular disease. According to the report of Teng et al. (2021), the increase of SFA content in human diet will directly lead to a significant increase in low-density lipoprotein and cholesterol, leading to an increase in the incidence of CVD diseases. The fatty acids deposited in beef mainly come from the diet. Chiofalo et al. (2020) found that compared with the grain diet, the C18:3n3 rich in pasture significantly increased the unsaturated fatty acid content in grass-fed beef. Dried olive cake fed to beef cattle significantly increased oleic acid content in beef. The study observed a decreasing trend in the content of saturated fatty acids in beef with an increase in alfalfa hay in the diet. This result is consistent with the findings of Du et al. (2022)'s study on feeding lambs with alfalfa hay using high, medium, and low concentrations. However, it is necessary to acknowledge the health trade-offs associated with higher saturated fatty acid (SFA) levels in the LAG group. While the LAG diet significantly improved certain meat quality parameters, lower SFA and higher unsaturated fatty acids (MUFA/PUFA) are generally preferred from a human health perspective to prevent cardiovascular diseases. We hypothesized that this is potentially due to the tannins present in alfalfa hay inhibiting rumen hydrogenation. However, it is important to acknowledge that the tannin content in alfalfa hay is generally relatively low. Because tannin concentrations in the diets of the different treatment groups were not directly measured in this study, this proposed mechanism remains a speculative hypothesis. Further *in vivo* studies incorporating direct measurements of dietary tannins and ruminal biohydrogenation are necessary to determine whether tannins contributed to the observed changes in fatty acid composition. Bryszak et al. (2019) reported that anthocyanins in berry seeds can change rumen hydrogenation and increase the concentration of unsaturated fatty acids in the rumen of cows. Polyphenols in grape pomace reduce rumen hydrogenation in lambs, thereby reducing the deposition of saturated fatty acids in lamb meat while increasing the concentration of unsaturated fatty acids. Tannins' inhibition of rumen hydrogenation can increase the concentration of unsaturated fatty acids in the rumen, and these fatty acids are eventually absorbed by the small intestine and deposited in muscle tissue. However, the impact of tannins from different sources on rumen hydrogenation varies, with acacia tannins having a negligible effect on rumen hydrogenation while papaya leaf tannins having a significant effect. Alfalfa tannins are known to have an inhibitory effect on rumen hydrogenation by hindering the breakdown of fats in the rumen and reducing the production of free fatty acids. This has been reported in several studies and is believed to have a positive impact on increasing the concentration of unsaturated fatty acids in rumen (Hoffmann et al., 2015). Furthermore, tannins in animal oral cavities can produce astringency by binding to salivary proteins, affecting the palatability of feed. Subsequently, tannins enter the digestive tract and can bind to various digestive enzymes, causing them to lose function, reducing the digestion rate of nutrients, increasing the retention time in the gastrointestinal tract, and ultimately reducing the feed intake of animals. It has been reported that tannins in the leaves of blackberry shrubs significantly reduce their palatability for goats, leading to a decrease in feed intake. Goats also selectively forage coarse old stems with low tannin content to avoid high tannin concentrations in new growth. In tropical and subtropical grassland areas, tannin content in shrubs and forage also directly affects the feeding behavior and dry matter intake (DMI) of livestock (Alm et al., 2002). Overall, these studies were consistent with the decrease in feed intake of beef cattle with increasing alfalfa hay content in the diet observed in this experiment, further supporting our hypothesis.

As the most important digestive organ of ruminants, the rumen depends on rumen microbes to cooperate to break down hard-to-use carbohydrates for their own needs and provide energy and high-quality bacterial protein to the host. As major members of the complex rumen ecosystem, bacteria play an integral role in decomposing diets. *Firmicutes* and *Bacteroidetes* were the dominant phyla in all groups at the taxonomic level, consistent with the findings of Fouts et al. (2012). Myer et al. (2015) investigated the variations in rumen microorganisms among beef cattle with different feed efficiencies and reported that the dominant bacteria in the rumen microorganisms of all beef cattle in the experiment were consistent with the ones identified in this study. *Firmicutes* can secrete corresponding enzymes to digest proteins, lipids, and cellulose, while *Bacteroidetes* are important bacteria in the rumen that decompose macromolecular nutrients into succinic acid, acetic acid, and lactic acid. Krajmalnik-Brown et al. (2012) have proposed that *Firmicutes* are more efficient at breaking down energy from diet than *Bacteroidetes* when ingested in diets with higher nutrient levels. This also explains why HAG has the highest abundance of *Firmicutes* in this study. *Actinobacteriota* are Gram-positive bacteria, which have unparalleled synthetic capacity to secrete a wide range of antibiotics. In this study, The relative abundance of Actinobacteriota decreased as the dietary proportion of alfalfa hay increased. This may be associated with plant secondary metabolites in alfalfa, including saponins, which could indirectly affect the rumen microbial community. However, since dietary saponin concentrations and rumen protozoal populations were not measured, this proposed mechanism remains hypothetical and requires further investigation. Increasing the proportion of dietary alfalfa hay led to an increase in the relative abundance of *Proteobacteria*, as observed in our study. *Proteobacteria* is a large phylum of gram-negative bacteria that encompasses many diverse species, including some pathogenic bacteria such as *Escherichia coli, Salmonella*, these bacteria typically release LPS into the surrounding environment (Rizzatti et al., 2017). As Byndloss et al. (2017) describe lack of butyrate-producing bacteria promotes the proliferation of *Proteobacteria*, this study observed that the concentration of VFA in the rumen of the HAG group was comparatively lower than the other groups, this also explains why the HAG abundance of Proteobacteria is higher than that of LAG and MAG. The dominant microbes in all four groups at the genus level were *Prevotella, Ruminococcus, Rikenellaceae_RC9_gut_group, UCG-005*, and *NK4A214_group*. *Prevotella* is considered one of the core genera in the rumen, which can carry out the decomposition and digestion of lignocellulose and the metabolism of carbohydrates. The colonization of *Prevotella* in the rumen begins about 1–2 weeks after the ruminant is born until it becomes a dominant bacterium in fiber-fed ruminants. *Prevotella* can secrete a variety of enzymes in the rumen, among which amylase is the most secreted, accounting for about 60% of the total secreted digestive enzymes. Prevotella bryantii 25A has also been evaluated as a probiotic for modulating ruminal fermentation in early-lactation dairy cows (Chiquette et al., 2008). Rodriguez et al. (2000) found that *25APrevotella* can quickly ferment starch and reduces lactic acid accumulation in the rumen by 90%, increasing the concentration of succinic and propionic acids. The other enzymes which from *Prevotella* are key enzymes in the rate of cellulose digestion, such as acetyl xylan esterase and ferulic acid esterase, which are more responsible for the degradation of hemicellulose than the digestion of cellulose (Dodd et al., 2010) The abundance of *Prevotella* in HAG was decreased, similar results were reported by Jami and Mizrahi (2012) *Rikenellaceae_RC9_gut_group* is a dominant genus within the *Rikenellaceae*, but its specific function has not been accurately reported yet. Researches show that *Rikenellaceae_RC9_gut_group* plays an important role in the fermentation of carbohydrates and proteins. Pitta et al. (2010) reported that *Rikenellaceae_RC9_gut_group* can degrade structural carbohydrates, with the gradual increase in the amount of alfalfa hay added, the abundance of *Rikenellaceae_RC9_gut_group* gradually decreased, which is inconsistent with previous reports, and the specific function of *Rikenellaceae_RC9_gut_group* needs further research. Previous studies reported that as the butyrate producer *Eubacterium_brachy_group* utilizes fiber in the rumen for their proliferation (Louis and Flint, 2017), the relative abundance of *Eubacterium_brachy_group* in LAG was significantly higher than that in other groups, the findings are also in agreement with the levels of butyric acid detected in the rumen. *norank_f__Eubacterium_coprostanoligenes_group* can transform cholesterol to coprostanol, coprostanol is poorly absorbed in the intestine, and most of it is excreted in the feces. In our study, the relative abundance of *norank_f__Eubacterium_coprostanoligenes_group* in LAG was much higher than that in HAG, but the specific reasons need to be further verified.

According to the description of the previous study, *Prevotella* is a Gram-negative bacterium, found in the human oral cavity, gut, and ruminant rumen. To meet the needs of growth and reproduction, *Prevotella* mainly fermented protein hydrolyzates to produce succinic acid and acetic acid, a minor quantity of isobutyric acid, and isovaleric acid. On the other hand, *Prevotella* is a highly prevalent genus of bacteria found in various regions of the rumen, which secretes enzymes that digest proteins, peptides, and starches, but it does not have the ability to degrade fibers, *Prevotella* plays an important role in the metabolism of hemicellulose, starch, xylan, lignan, and pectin (Purushe et al., 2010). In our study, as the isobutyric productor, *Prevotella* were positively correlated to Isobutyric acid. Prior research has indicated that *Rikenellaceae_RC9_gut_group* is a prevalent microbial community in the rumen of cattle, dairy cows, yak and even growth-retarded yak, these results are also similar to this study (Ma et al., 2020), at the same time, we found that *Rikenellaceae_RC9_gut_group* abundance decreased with increasing dietary alfalfa content, and *Rikenellaceae_RC9_gut_group* were positively correlated to Acetate acid, the specific functions of *Rikenellaceae_RC9_gut_group* are still unclear, the specific reasons need to be further study. When carbohydrates are fermented, several numerically and functionally rumen bacteria create succinate; nevertheless, succinate is rarely found in quantifiable concentrations in rumina because it is quickly converted to propionate, the significance of the organism *Succiniclasticum* in rumen is demonstrated by the fact that it specializes in fermenting succinate and quantitatively converting it to propionate. On the other hand, propionic acid is an important precursor of glucose. Previous studies showed that glucose had a detrimental impact on acarbose synthesis while maltose containing had a positive impact. This also explains why *Succiniclasticum* were significantly negatively correlated with Acarbose and validamycin biosynthesis in this study. Several studies have demonstrated the reciprocal connection between *Fibrobacter* and *Treponema, Fibrobacter* and *Treponema* have a symbiotic relationship in the rumen, where *Fibrobacter* relies on *Treponema* to break down complex hemicellulose structures and make fiber components more accessible, in return, *Fibrobacter* provides *Treponema* with cellulose hydrolysates (Xie et al., 2018). Several monosaccharide and polysaccharide metabolic pathways showed significant positive correlations with Treponema in this study, recent research has shown that *Treponema*, represented by *T. saccharophilum*, degrades pectin anaerobically through the Entner-Doudoroff route, and the only VFA generated throughout the fermentation process is acetate (Liu et al., 2015). Perhaps there are still many reciprocal connections between metabolism-related microbiota and *Treponema* that have not been found, this indicates that more research is required to fully understand the underlying mechanisms involved. A limitation of this study is the relatively small sample size used for rumen fermentation and microbiome analyses, with only four independent samples per treatment (*n* = 4). This limited sample size may reduce statistical power and increase the influence of inter-animal variation. Therefore, these findings should be interpreted with caution and require further validation in studies with larger sample sizes.

## 5 Conclusion

In conclusion, 11.57% alfalfa hay in the diet is the most suitable ratio for beef production. This diet was found to improve the final weight and ADFI, significantly reduces beef shear forces, significantly increased the abundance of *Eubacterium_brachy_group, norank_f__Eubacterium_coprostanoligenes_group* in the rumen. Additionally, this study provides a scientific basis for the production of high-quality beef that meets the demand for premium functional animal products. The findings can serve as a theoretical and practical foundation for the industry.

## Data Availability

The 16S rRNA gene sequencing data generated in this study are available in the NCBI Sequence Read Archive (SRA) under BioProject accession number PRJNA973793. Other data supporting the conclusions of this article are included in the article and its Supplementary Material. Further inquiries can be directed to the corresponding author.
