# Supplementary material for: Improving beef quality through rumen microbial modulation by varying alfalfa hay proportions in the diet
Source: Front Microbiol. 2026 Sep 29;17:1961267. doi: 10.3389/fmicb.2026.1961267 (PMC13658148; doi:10.3389/fmicb.2026.1961267)
Supplement: Supplementary file 1 [file Table_1.docx]

Supplementary Material

# 1 Supplementary Tables

**Table S1** Diet composition and nutritional components （dry matter basis）

| Items | LAG | MAG | HAG |
| --- | --- | --- | --- |
| Ingredient（%） |  |  |  |
| Corn Silage | 34.71 | 25.45 | 16.20 |
| Alfalfa Hay | 11.57 | 20.83 | 30.09 |
| Concentrate^1^ | 38.76 | 38.76 | 38.76 |
| Bean dregs | 3.33 | 3.33 | 3.33 |
| Distilled grain | 11.63 | 11.63 | 11.63 |
| Total | 100.00 | 100.00 | 100.00 |
| Nutritive level^2^ |  |  |  |
| NEmf/（MJ/Kg） | 6.473 | 6.483 | 6.493 |
| CP | 11.017 | 11.796 | 12.5763 |
| NDF | 25.309 | 26.440 | 27.570 |
| ADF | 18.239 | 18.644 | 19.05 |
| Ca% | 0.583 | 0.762 | 0.940 |
| P% | 0.166 | 0.190 | 0.214 |

^1^ Ingredients of concentrate were 60% corn,10% DDGS,10% soybean meal,13% wheat bran,1%limestone,2% NaHCO_3_,4% premix. The premix provided the following per kg of diets：Fe 1 200 mg, Zn 450 mg, Cu 150 mg, Se 5 mg, I 15 mg, Co 4 mg, VA 150 000 IU, VD3 50 000 IU, VE 500 mg.

^2^ Nutrient levels were calculated values.NEmf was calculated accord to Feeding Standard of Beef Cattle (NY/T 815-2004), while the others were measured values.
